# Priority setting in dementia palliative care research for people living with dementia and carers: A mixed methods consensus study

**DOI:** 10.1177/26323524251392625

**Published:** 2025-11-21

**Authors:** Ping Guo, Nikolaos Efstathiou, Cara Bailey, Peymané Adab, Jon Glasby

**Affiliations:** 1 Department of Nursing and Midwifery, School of Health Sciences, College of Medicine and Health, University of Birmingham, UK; 2 Department of Applied Health Research, School of Health Sciences, College of Medicine and Health, University of Birmingham, UK; 3 Department of Social Work and Social Care, School of Social Policy and Society, College of Social Sciences, University of Birmingham, UK

**Keywords:** dementia, palliative care, end-of-life care, priorities, consensus building, patient and public involvement and engagement

## Abstract

**Background:**

People living with dementia have complex needs, which will eventually lead to increasing demand for palliative care. However, there is limited evidence on the priority setting in palliative care specifically for people with dementia and carers.

**Objectives:**

This study aimed to identify priorities for dementia palliative care research in the West Midlands of England.

**Design:**

A mixed methods consensus study.

**Methods:**

Following a rapid review of recommendations on priorities for palliative or dementia care research, we identified 54 priorities across 11 domains, which were discussed with 20 key stakeholders (clinicians, researchers, local government representatives, and experts by experience) at a stakeholder consultation workshop. Then a list of 45 topics was developed and informed the development of an online cross-sectional survey. Consensus-building techniques were used where these topics were rated for importance and ranked to indicate top priorities for dementia palliative care in the region. Descriptive statistics was used to analyse the survey quantitative data and content analysis for free text responses.

**Results:**

Forty-three stakeholders completed the online survey. The top 10 priorities include symptom assessment and management (e.g. pain, nausea, vomiting, acute, and/or chronic breathlessness); staff training and cultural competence; treatment and support for distress and delirium; care for people with advanced dementia at the end of life and their carers in all settings; the needs of young people with dementia and their carers; food and nutrition (e.g. difficulty in swallowing); supporting carers of people with dementia living at home; the needs of people who live alone; advance care planning and other approaches incorporating individual preferences; and home care and coordination of services.

**Conclusion:**

Our results suggest the top priorities for dementia palliative care research, thus informing future practice, policy, and research.

## Introduction

Dementia is a growing challenge globally with the highest proportional increase of 6 million people (264% increase) between 2016 and 2060, based on population projections.^1^ Care systems for older people with dementia are inadequate, inequitable, and unable to address their growing needs.^2,3^ In the United Kingdom, long-term care is under-resourced and lacks integration with healthcare services, which compromises continuity of care.^4,5^ Moreover, older people with dementia typically live with complex needs associated with multimorbidity. This requires the provision of holistic and person-centred care with comprehensive assessments and care planning to identify, monitor, and manage underlying conditions and symptoms.^6^ Above all, many individuals with dementia and their families report insufficient support for living fulfilling lives and making end-of-life decisions, often feeling denied choice and lack of control over their care and lives, which can lead to feelings of frustration and powerlessness.^7,8^

There are currently 850,000 people living with dementia in the United Kingdom. This is set to rise to 1.6 million by 2040.^9,10^ Some UK research investigates inequities in access to long-term care for older people with cognitive impairment.^11–13^ These show high unmet needs in terms of dementia care support, with lower access to health and long-term care services, and greater reliance on family care.^14–16^ Many families describe such care as psychologically, physically, and financially demanding, while formal long-term care services are often unaffordable, especially for families from lower socio-economic groups.^17–19^ At its best, caring ‘for’ someone comes out of caring about someone, and there is a real risk that families have no choice but to provide extensive unpaid care due to the high cost and lack of alternative care options, irrespective of what the person or the family might want. This situation has caused significant strain on the health, financial stability, and work–life balance of many unpaid carers.^20,21^

Dementia is the leading cause of death in the United Kingdom and causes complex cognitive and behavioural symptoms. It contributes to a significant proportion of disability among older people, with profound political, economic, and social consequences. The total economic cost of dementia is projected to increase from £23.0 billion in 2015 to £80.1 billion in 2040, and average cost is projected to increase from £35,100 per person per year in 2015 to £58,900 per person per year in 2040.^22^ In the context of rapid population aging, increased prevalence rates of dementia will lead to heightened demand for palliative care. Palliative care is a neglected area of research in cognitive impairments.^23^ People with severe cognitive impairments often live with multiple long-standing medical conditions associated with advancing age. This increases the risk of distressing symptoms and the complexity of care needs.^24^ Current services aren’t sufficiently inclusive of the needs of people with dementia and their families, and don’t do enough to support people to have a good life, or indeed a good death (i.e. putting the onus on systems and services to get more person-centred care).^25,26^ In the United Kingdom, more than one million people with severe cognitive impairments face additional complications at the end of their lives,^27^ and three in five (61%) of people affected by dementia did not feel they had received enough support in the last 12 months.^28^ Hence, the integration of palliative care into dementia care is essential for delivering equitable, effective, and person-centred care for this population and carers.

However, a lack of consensus on dementia palliative care priorities may lead to systemic inefficiencies, fragmented care and support, and unmet needs for individuals with dementia and their families.^29^ Disagreements on key aspects like person-centred care plans, advance care planning, and criteria for decision-marking after capacity loss contribute to gaps in care, such as inconsistent diagnosis processes, insufficient post-diagnosis support, fragmented care pathways, and inadequate workforce training.^30^ This ultimately exacerbates the societal burden of dementia through higher care costs, poor quality of life, and reduced independence for affected individuals.^31^ Furthermore, little is known about palliative care needs in people with moderate and severe cognitive impairments who live at home or in care homes, and how these needs change with disease progression. Professionals often lack the skills to manage the challenges presented when someone with cognitive impairments is nearing the end of life.^32^

Although palliative care is recognised as a basic human right and an essential health service under Universal Health Coverage,^33^ ensuring access and tackling inequities in palliative care is a challenge for meeting the needs of people living with dementia and their families. Palliative care services are patchy, inequitable, and not always age appropriate, which then poses challenges, with rapid deterioration and a lack of time to prepare for death.^34^ Incorporating diverse stakeholder perspectives to identify and address the most pressing needs in dementia palliative care is essential to ensure interventions and services are relevant, effective, and address the actual needs of people living with dementia, carers, and healthcare professionals.^35^ This approach increases the chances of successful implementation of new interventions and services by aligning them with lived experiences and preferences, leading to better patient outcomes, improved care quality, and more innovative solutions to care.^36^

Priority setting in dementia palliative care research is crucial for reducing preventable suffering and improving the quality of life and end-of-life care for individuals with dementia. This involves recognising what matters to people living with dementia and their families, understanding their needs, preferences, and goals of care, and implementing culturally appropriate interventions that address these priorities.^37,38^ Previous studies have mostly focused on priorities for palliative care in general.^39,40^ However, there is limited evidence on the priority setting in palliative care research specifically for people with dementia and carers. Given that the West Midlands is the second most diverse region in the United Kingdom (in terms of ethnicity, socio-economic status, and a mix of urban and rural authorities) and has the highest dementia prevalence, this study aimed to identify priorities for dementia palliative care research in the West Midlands of England through stakeholder consultation and engagement. The results from this study suggest the top priorities for dementia palliative care research, providing a resource for researchers and clinicians seeking solutions to providing optimal palliative care for individuals living with dementia and their carers, thus informing future practice, policy, and research.

## Methods

This mixed methods consensus study was conducted in three stages, utilising consensus-building techniques: (1) identification and collection of relevant priorities, (2) stakeholder engagement and consultation using a nominal group technique, and (3) prioritisation of topics through an online cross-sectional survey. The overall study design was informed by the MORECare Transparent Expert Consultation approach to generating and ranking priorities to ascertain consensus.^41^ The Strengthening the Reporting of Observational Studies in Epidemiology (STROBE) guidelines^42^ for cross-sectional studies (Supplemental File 1) and reporting guideline for priority setting of health research (REPRISE) were followed.^43^

### Stage 1: Identification and collection of relevant priorities

Existing recommendations on priorities for either palliative or dementia care research were reviewed to identify and collect pre-existing priorities that had been developed with international experts, including academics and healthcare professionals.^23,44–46^ Two researchers (P.G. and N.E.) independently reviewed the key recommendations, and identified and collected priorities, which were then cross-checked by the wider project team and UK experts in the field to ensure the generation of a comprehensive list of priorities for discussion and consultation with stakeholders in Stage 2.

### Stage 2: Stakeholder engagement and consultation

Stakeholders from social care, primary care and public health, academics, clinicians, local government representatives, and experts by experience (people with dementia and family carers) were invited by email to attend a virtual workshop on 9 November 2022. The stakeholders, mainly from Birmingham and the West Midlands region, were members of a newly developed dementia palliative care hub, based on a palliative care research network (https://www.birmingham.ac.uk/schools/nursing/research/brhumb).

The virtual workshop was organised to discuss the Stage 1 findings, using nominal group techniques.^47^ Six steps were included: (1) Using an opening statement to set the scene, for example, warm welcome followed by an overview of the study, setting out the aims of this workshop, and presenting the priorities arising from the review (which were sent via emails for stakeholders to read prior to the workshop). (2) A silent generation of ideas: Stakeholders were invited to work silently and independently by thinking about the following question and recording responses in brief phrases or statements: what do you think are the priorities in dementia palliative care research that have been missed from the list? (3) Sharing ideas and feedback using a ‘round robin’ technique (without debate at this point). (4) Four concurrent breakout rooms were used to discuss and identify missing priorities, which were facilitated by a core project member per group to ensure that all participants had an opportunity to contribute, and all the ideas were recorded. (5) Clarification of ideas through a whole group discussion aimed at ensuring that all ideas, views, and experiences presented were understood by everyone. This step also provided an opportunity for members to express their understanding of the logic and the relative importance of the priorities. (6) Closing remarks and invitation to respond to a follow-up online survey for Stage 3.

All group discussion notes were recorded and collated after the workshop for further analysis. The main points were compared and discussed to ensure adequate synthesis of similarities and differences in the views expressed. At this stage, duplicates were removed, and similar priorities were amalgamated.

### Stage 3: Prioritisation

A list of 45 priorities was derived for further refinement and ranking in an online survey using Jisc Online Surveys (v1, Bristol) with an introduction page at the beginning. The priorities were presented in random order. The survey was piloted for face validity by two researchers (P.G. and N.E.) prior to the survey going live. Email invitations were sent out including a description of the study and the link to access the survey. Consent was assumed for any participant who chose to complete and return the survey. The survey was launched on 20 April 2023 and remained open for 6 weeks until 31 May 2023. Two reminders were sent.

The online survey was available for people who joined the workshop, those in the BRHUmB palliative care network mentioned above, and national experts in palliative care (who were reminded to advise on the priorities for the West Midlands region based on their experience and expertise). All the key stakeholders who had lived experience of dementia or experience in providing care for people with dementia and academics/researchers in this field were invited for participation. Participants were asked to rate the importance of each priority on a 5-point Likert scale (where 5 indicated extremely important; 4 very important; 3 important; 2 not very important; 1 not at all important). Apart from the rating scales, participants were also provided the opportunity to comment on the phrasing and clarity of each priority in a free text box and to suggest any additional comments at the end of the survey (Supplemental File 2).

To determine the degree of consensus, Delphi technique principles were used.^48–50^ Descriptive statistics were used to analyse the survey quantitative data (e.g. median to capture central tendency and interquartile range – IQR for dispersion) and content analysis for free text responses.^51,52^ Consensus was deemed to have been reached for priorities that received aggregated responses with an IQR ⩽1 and a median ⩾4, or for those priorities that were scored 4 or 5 by at least 70% participants. Priorities reaching this consensus were included in the final set of top priorities.

### Ethics

This study was undertaken as part of the wider priority setting exercise for palliative care research, which has been published in a separate paper.^53^ It was reviewed by the relevant ethics committee (the University of Birmingham Research Governance and Integrity Committee), and ethics approval was waived as this was not required as public and patient involvement and engagement activities. However, all the workshop and survey participants were informed that data would be anonymised, and results would be published. Verbal consent was obtained prior to the workshop, and the responses to the online survey were considered as implied consent. No personal or sensitive data were collected, and the principles of anonymity and confidentiality were upheld throughout.

## Results

The review of existing recommendations (Stage 1) identified 54 relevant priorities. These priorities were categorised into 11 domains including applicability of palliative care (*n* = 7); person-centred care, communication and shared decision-making (*n* = 8); setting care goals and advance planning (*n* = 3); continuity of care (*n* = 4); prognostication and timely recognition of dying (*n* = 2); avoiding overly aggressive, burdensome or futile treatment (*n* = 1); optimal treatment of symptoms and providing comfort (*n* = 13); psychosocial and spiritual support (*n* = 5); family care and involvement (*n* = 5); education of the healthcare teams (*n* = 2); and societal and ethical issues (*n* = 4).

Twenty key stakeholders attended the virtual workshop (Stage 2) to discuss the 54 priorities. They were clinicians (*n* = 7), academics/researchers (*n* = 8), local government representatives (*n* = 2) and experts by experience (*n* = 3). The following five questions guided discussion: (1) Are the priorities clear and easy to understand? (2) Are the priorities focused (e.g. some may be too broad to be researched and need refinement, so ask the question about ‘is its scope well-defined and achievable’)? (3) Are the priorities substantively relevant and important (e.g. ask the questions about ‘What makes it interesting and worthy of research’)? (4) What do you think are the research priorities in dementia palliative care that have been missed from the list? (5) What do you think the West Midlands of England could contribute to the field of dementia palliative care?

After the analysis of workshop discussion, the wordings in some priorities were revised to improve clarity and accuracy, and to ensure the use of dementia friendly language. ‘Dementia patients’ were replaced with ‘people with dementia’, and ‘challenging behaviours’ changed to ‘behaviours which other people find challenging to manage’. A few priorities were refined or grouped as they were too broad to be researched or addressed similar topics. In addition, the priorities which had been missed from the review were added including the importance of outcome measurement, integrated dementia palliative care for disadvantaged populations such as LGBTQ+ communities.

As a result, 45 priorities were used to form the basis for the subsequent online survey (Stage 3). Forty-three participants completed the online survey. Their characteristics are shown in Table 1. There were more female (*n* = 34) than male participants (*n* = 8). Seventy-four percent (*n* = 32) of participants were white British. Our survey participants were from different backgrounds including people living with dementia, carers, academics, researchers, and healthcare providers from different profession groups and working across care settings.

**Table 1.** Participants’ characteristics (*n* = 43).

Characteristics	*n* (%)
Age groups	
25–34 years old	7 (16.3)
35–44 years old	7 (16.3)
45–54 years old	12 (27.9)
55–64 years old	12 (27.9)
65 years old and over	5 (11.6)
Gender	
Male	8 (18.6)
Female	34 (79.1)
Prefer not to say	1 (2.3)
Ethnic groups	
White British	32 (74.4)
African	3 (7)
Indian	2 (4.7)
White Irish	1 (2.3)
Other White	2 (5)
Other mixed background	1 (2.3)
Other (e.g. Middle Eastern)	2 (4.7)
Experience in providing care for people with dementia	
Less than 30% of my time I care for person(s) living with dementia	14 (32.6)
30%–60% of my time I care for person(s) living with dementia	8 (18.6)
More than 60% of my time I care for person(s) living with dementia	4 (9.3)
Not applicable (e.g. currently no dementia caring responsibilities)	17 (39.5)
Which of the following best describes you? (Select all that apply)	
A person living with dementia	1 (2.3)
A carer or family member or partner or friend of a person living with dementia	8 (18.6)
A member of the public who has an interest in the subject	8 (18.6)
An academic or researcher in this area	13 (30.2)
A professional working with people living with dementia	21 (48.8)
Which region do you work?	
West Midlands	34 (79.1)
London	5 (11.6)
North East	2 (4.7)
North West	1 (2.3)
East Midlands	1 (2.3)
Role/expertise	
Expert by experience (e.g. people with dementia, carers)	13 (30.2)
Palliative care specialist	9 (20.9)
Registered or enrolled nurse	5 (11.6)
Nurse practitioner	4 (9.3)
Assistant in nursing or aged care worker	2 (4.7)
Pharmacist	1 (2.3)
Geriatrician	1 (2.3)
Researcher	3 (7.0)
Other (e.g. psychologist, community dementia service provider, public health, allied health professional, integrated care board)	5 (11.6)
Setting (*n* = 30; select all that apply)	
Primary care/community	15 (50)
Hospital	8 (26.7)
Hospice	7 (23.3)
Residential aged care facility	3 (10)
Outpatient/outreach clinics	1 (3.3)
University or research institute	4 (13.3)
Other (e.g. community hospital, community voluntary sector, mental health commissioning and system partnership, and integrated care board)	5 (16.7)
How long have you worked in your current profession? (*n* = 30)	
<1 year	1 (3)
1–5 years	4 (13)
6–10 years	5 (17)
11–15 years	2 (7)
16–20 years	12 (40)
More than 20 years	6 (20)

### Prioritisation

The degree of consensus of all the 45 priorities is displayed in Supplemental File 3. Five items (1, 14, 5, 3, and 2) reached both predetermined consensus criteria (IQR ⩽1 and median ⩾4; at least 70% of participants who scored 4 or 5), which suggests strong agreement with the importance of these priorities. Based on *n* (%) of participants who scored 4 or 5, all the priorities were ranked. The top ten priorities (Table 2) related to symptom assessment and management (e.g. pain, nausea, vomiting, acute, and/or chronic breathlessness; No. 1); staff training and cultural competence (No. 14); treatment and support for distress and delirium (Nos. 5 and 6); care for people with advanced dementia at the end of life and their carers in all settings (No. 3); the needs of young people with dementia and their carers (No. 27); food and nutrition (e.g. difficulty in swallowing; No. 2); supporting carers of people with dementia living at home (No. 10); the needs of people who live alone (No. 17); advance care planning and other approaches incorporating individual preferences (No. 32); and home care and coordination of services (No. 39).

**Table 2.** Top 10 priorities from the online survey (*n* = 43).

Item number and priorities	Median	IQR	*n* (%) of participants who scored 4 or 5	Ranking
1. What are the best ways to assess and treat pain and other symptoms (e.g. nausea, vomiting, acute, and/or chronic breathlessness) in people at the end of life with communication and/or cognitive difficulties, due to dementia?	4.5	1	88.4	1
2. What are the most effective ways to encourage people with dementia to eat, drink, and maintain nutritional intake? What are the best ways to manage the problems associated with difficulty in swallowing, including drooling and excessive salivation, for patients with dementia who are at the end of their life?	4.5	1	74.4	6
3. What is the best way to care for people with advanced dementia (with or without other illnesses) at the end of life? What interventions are of most benefit in improving the quality of life for people with advanced dementia and their carers in all settings?	4	1	79.1	4
5. How can distress that is not related to pain be best assessed and managed in palliative patients with dementia that affect communication? What are the best approaches to reducing distress such as life story, pictures, reminiscence? How can sensory and environmental triggers be identified for people with dementia, and what education around coping strategies could support and reduce distress?	4.5	1	81.4	3
6. What are the best ways to diagnose and treat delirium, agitation, distress, and restlessness in people with dementia at the end of life? Which sedative drugs (such as midazolam, haloperidol, and levomepromazine) are most beneficial and best in terms of side effects? Do these drugs have an effect on other symptoms?	4	2	69.8	9
10. What are the most effective ways of supporting carers of people with dementia living at home?	4	1.5	74.4	6
14. How can it be ensured that staff, including healthcare assistants, are adequately trained to deliver palliative care for people with dementia, no matter where the care is being delivered? Does increasing the number of staff increase the quality of care provided in all settings? To what extent does funding affect these issues? How can cultural competence of palliative care staff be improved?	4	1	83.7	2
17. How can people with dementia who live alone and do not have any friends or family nearby receive adequate palliative care, particularly if they wish to stay in their homes?	3.5	2	72.1	8
27. What are the needs and priorities of palliative and end-of-life care for young persons with dementia when diagnosed at working age and their carers?	3.5	1.5	74.5	5
32. What are the benefits of advance care planning and other approaches to listening to and incorporating the preferences of people with dementia (or listening to carers, considering capabilities of patients)? Who should implement this and when?	2.5	1.5	69.8	9
39. What are the best models and approaches to providing palliative care for people with dementia at home, and how can home care be maintained as long as possible? Does good coordination of services affect this?	4	2	69.8	9

IQR, interquartile range.

### Free text analysis

The survey participants provided insights into the priorities rated and shared additional information to explain or justify their ratings, underlining the importance of offering space for comments. Pain was considered as one of the most common symptoms that people with dementia experience. Our survey participants felt that pain was poorly recognised and undertreated in dementia. Pain and other symptoms were ‘*the most important things’ affecting quality of life and functioning of people with cognitive impairment*’. However, articulating these symptoms was not straightforward and could be frustrating for them. As dementia progresses, the person’s ability to communicate their needs becomes more difficult. Signs and behaviours often have to overrule words, for example, the person with dementia who says she has no pain. She may have lost the capacity to understand the question or to label the pain. Thus, finding ways of assessing and treating these symptoms that don’t require asking lots of questions is important. In addition, people felt that pain and other symptoms not recognised and addressed could be central drivers of distress. They also suggested that ‘*emphasis may be best placed on holistic assessment and optimal management of symptoms*’.

The participants regarded cultural aspect of care as a priority. It was considered that dementia palliative care may not have been designed with people from certain cultures and therefore they may miss out on essential support and experience inequities particularly for those from minority ethnic communities. Cultural competence and confidence of staff are important, and multicultural teaching and explanations should always be provided for staff working in multicultural environments. Experts considered that ‘*increasing the number of staff alone was not that important, what was important would be the number and the mix of skills and expertise*’. Adequate staff training and support could help prevent burnout and therefore maintain better quality of care for all and staff engagement and satisfaction. Staff across all health professions would benefit from further training with cultural differences as ‘*this is important for patients and their families and there is only one chance to get it right*’, as clearly stated by one of our survey participants.

The participants agreed that the integration of palliative care into dementia practice was essential to provide support and comfort to everyone involved including carers. People commented that where someone was going into a nursing (care) home, ‘*it would be beneficial for the nursing (care) home to know the persons’ wishes before they came in order to determine the best way to care for them*’. Advance care planning could help with this. As one participant highlighted ‘*There may be individual, personalised things that may improve the quality of life, for example knowing what soothes a person (e.g., music, television shows, spiritual support, or having someone around)*’. In addition, some participants recognised that young people with dementia when diagnosed at working age and their carers were ‘*a very neglected group in terms of palliative care provision*’. Their age-appropriate needs would need to be explored, acknowledged, and addressed, which was an under-researched area and deserved attention.

The participants shared a consensus about the importance and need to ensure continuity of support for carers throughout the whole journey of caring for someone living with dementia and access to extra support towards the end stage of life. Carers were seen as the main providers of care for adults with dementia at home. The problem identified was not a lack of evidence on what was effective. People felt that ‘*what had been missing was the policy and funding priority to implement effective interventions*’. The NHS Long Term Plan prioritised carers – but this was mainly focused on assessment. Palliative and end-of-life care for carers supporting people with dementia was considered ‘*very important as they were often having to co-ordinate care and made difficult decisions*’. A variety of sources (e.g. online, help line, GP) were recommended for use to ensure that carers are safe and supported.

## Discussion

### Main findings

Key stakeholders with relevant experience of healthcare systems were engaged to provide insights into the priorities within dementia palliative care research for the West Midlands region. Top 10 priorities were identified including symptom assessment and management (e.g. pain, nausea, vomiting, acute, and/or chronic breathlessness); staff training and cultural competence; treatment and support for distress and delirium; care for people with advanced dementia at the end of life and their carers in all settings; the needs of young people with dementia and their carers; food and nutrition (e.g. difficulty in swallowing); supporting carers of people with dementia living at home; the needs of people who live alone; advance care planning and other approaches incorporating individual preferences; and home care and coordination of services. Free text comments were analysed to further explain why these priorities were considered as highly important.

Symptom assessment and management has been ranked as a top one priority in our work, which signifies that it requires immediate attention and is placed above all other priorities. However, pain and other symptoms are often overlooked or undertreated in people with dementia, leading to reduced quality of life and potential complications.^54^ Communication barriers could be influencing factors, particularly when people with dementia have difficulties expressing their pain or reporting symptoms due to cognitive impairment, verbal communication difficulties, or a lack of awareness of pain.^55,56^ In addition, carers and healthcare professionals experience challenges in identifying pain in people with dementia due to the limited pain assessment tools specifically designed for this group. They often underestimate the level of pain and other symptoms as they may prioritise addressing increased distress and worsening behavioural symptoms (e.g. agitation, anxiety), but overlook the underlying pain that could be contributing to those behaviours.^57^ It is crucial to train carers and healthcare professionals about pain and other symptom assessment, management strategies, and the importance of recognising and addressing pain and other symptoms in individuals with dementia.^58,59^ Non-pharmacological approaches to manage pain such as music therapy, massage, and repositioning are often underutilised and should be promoted. Evidence shows these holistic approaches can be effective in reducing both physical and psychological aspects of pain and improving overall well-being.^60–62^

Research documents the presence of stigma and discrimination in the lived experience of dementia, which could be compounded by culture, race, and immigration status.^63^ Consistent with other research, most participants in this study described the concerns of inadequate cultural competence such as a lack of cultural sensitivity and awareness. This can lead to inaccurate diagnoses, ineffective treatment plans, and increased disparities in health outcomes for disadvantaged populations including people from minority ethnic communities^64^ and younger people with dementia.^65^ Inadequate cultural competence may also create communication barriers among people with dementia, carers, and healthcare providers who come from different cultures, limiting access to and use of essential services like palliative care.^66^ Our work reinforces the evidence of persistent inequities in accessing health and social care faced by minority communities – who might well be disadvantaged because of mainstream and specialist services not being specifically designed to meet their unique needs.^13^ A scoping review highlighted the need of developing an inclusive model of culturally appropriate care to people with dementia from culturally diverse backgrounds in the nursing home context.^67^ To ensure an equitable access to dementia palliative care services among disadvantaged populations, an urgent call to action is needed to involve adequately trained intercultural mediators, and ensure quality training in cultural competence for health staff and a commitment to increase workforce diversity.

While the importance of person-centred approach is increasingly recognised to meet the needs of people with dementia and their carers, there is little guidance on how person-centred care can be effectively implemented and researched in the context of dementia palliative care.^68,69^ Person-centred care involves understanding and incorporating individual needs and preferences, treating them with dignity, and involving them in decision-making,^70,71^ which was also prioritised by diverse stakeholders in our work. Developing individualised care plans (e.g. advance care planning) based on the specific needs and preferences of every individual living with dementia is essential.^72^ Healthcare professionals should work together with and for people with dementia and their carers to co-create environments that are personalised and supportive, and co-design integrated health and social care services regardless of their care needs, financial status, or where they live and receive care.^73^

### Strengths and limitations

The results of this study shed light on which topics key stakeholders regard as priorities for dementia palliative care research. The recommendations help to focus future research into the areas that matter most to the people involved, addressing an important gap in the literature. This is the first attempt to bring together a range of expertise and experience to discuss this topic and set priorities for the West Midlands region in the United Kingdom. The results may not apply to other contexts and countries, although they could provide preliminary priorities as a basis for a similar study elsewhere. The survey was pilot tested for face validity by project team members prior to the survey going live. Evaluating the reliability and validity of the survey tool with a wider range of our target population would have strengthened our work. Furthermore, analyses of free text responses to open-ended questions remain time and resource-intensive; therefore, are rarely performed and published, and the additional insights within this type of data are often underutilised.^74^ Indeed, the content analysis of free text comments in our survey was a strength, which served various purposes, including the provision of deeper insights on specific priorities, identification of issues that closed-ended questions might not reveal, and suggestions about quality improvement initiatives.

The results indicate the views of a small sample of key stakeholders including people with dementia and carers, healthcare professionals, academics, people from local government, and selected national experts, but predominantly from Birmingham and the West Midlands. Sample size calculation was not conducted; therefore, the results should be interpreted with caution because the small sample may have led to underpowered findings. Although group diversity was a strength, greater representation of people with dementia and carers (e.g. people from the LGBTQ+ community, from minority communities) would have enriched the group. Whilst it would not be realistic to include all marginalised groups in society, under-represented groups within dementia should be given a voice to ensure a wider range of perspectives. Birmingham has become the first ‘super-diverse’ city in the United Kingdom with 51% of population who are from black, Asian, or minority ethnic backgrounds.^75^ In response to these challenges and based on our results, a qualitative interview study (ethical approval number: ERN_2097-May2024) is underway to explore inequities and challenges faced by people with dementia and carers from minority ethnic communities and possible solutions.

### Implications for practice and policy

This study suggests that there was overall good agreement on the importance of recommendations for dementia palliative care research amongst key stakeholders. The prioritisation of symptom management and staff training for all working in health and social care and other support systems accords with the international evidence. Improving culturally appropriate and person-centred care for people living with dementia and their carers is warranted. Experts also prioritised the implementation of integrated dementia palliative care across different care settings. However, despite the endorsement by policymakers, specific palliative care policies and their implementation often remain underdeveloped globally, impacting access to essential services and care for individuals with serious illnesses including dementia.^76–78^ Greater inclusion of these priority topics is urgently required in national policies and health plans to address the needs of people with dementia and carers.

## Conclusion

Our results suggest the top priorities for dementia palliative care research, and these top priorities achieved consensus among a group of key stakeholders. Identifying these priorities is important to inform future practice, policy, and research.

## Supplemental Material

sj-docx-1-pcr-10.1177_26323524251392625 – Supplemental material for Priority setting in dementia palliative care research for people living with dementia and carers: A mixed methods consensus study Supplemental material, sj-docx-1-pcr-10.1177_26323524251392625 for Priority setting in dementia palliative care research for people living with dementia and carers: A mixed methods consensus study by Ping Guo, Nikolaos Efstathiou, Cara Bailey, Peymané Adab and Jon Glasby in Palliative Care and Social Practice

sj-docx-2-pcr-10.1177_26323524251392625 – Supplemental material for Priority setting in dementia palliative care research for people living with dementia and carers: A mixed methods consensus study Supplemental material, sj-docx-2-pcr-10.1177_26323524251392625 for Priority setting in dementia palliative care research for people living with dementia and carers: A mixed methods consensus study by Ping Guo, Nikolaos Efstathiou, Cara Bailey, Peymané Adab and Jon Glasby in Palliative Care and Social Practice

sj-docx-3-pcr-10.1177_26323524251392625 – Supplemental material for Priority setting in dementia palliative care research for people living with dementia and carers: A mixed methods consensus study Supplemental material, sj-docx-3-pcr-10.1177_26323524251392625 for Priority setting in dementia palliative care research for people living with dementia and carers: A mixed methods consensus study by Ping Guo, Nikolaos Efstathiou, Cara Bailey, Peymané Adab and Jon Glasby in Palliative Care and Social Practice
