# Supplementary material for: Priority setting in dementia palliative care research for people living with dementia and carers: A mixed methods consensus study
Source: Palliat Care Soc Pract. 2025 Nov 21;19:26323524251392625. doi: 10.1177/26323524251392625 (PMC12640447; doi:10.1177/26323524251392625)
Supplement: sj-docx-3-pcr-10.1177_26323524251392625 – Supplemental material for Priority setting in dementia palliative care research for people living with dementia and carers: A mixed methods consensus study [file sj-docx-3-pcr-10.1177_26323524251392625.docx]

**Supplementary file 3.**

**Results from the online survey (n=43) - the top ten priorities highlighted in grey**

| **Item number and priorities** | **Median** | **IQR** | **n (%) of participants who scored 4 or 5** | **Ranking** |
| --- | --- | --- | --- | --- |
| 1. What are the best ways to assess and treat pain and other symptoms (e.g., nausea, vomiting, acute and/or chronic breathlessness) in people at the end of life with communication and/or cognitive difficulties, due to dementia? | 4.5 | 1 | 88.4% | 1 |
| 2. What are the most effective ways to encourage people with dementia to eat, drink and maintain nutritional intake? What are the best ways to manage the problems associated with difficulty in swallowing, including drooling and excessive salivation, for patients with dementia who are at the end of their life? | 4.5 | 1 | 74.4% | 6 |
| 3. What is the best way to care for people with advanced dementia (with or without other illnesses) at the end of life? What interventions are of most benefit in improving the quality of life for people with advanced dementia and their carers in all settings? | 4 | 1 | 79.1% | 4 |
| 4. What are the most effective management approaches and models of care for people living with  dementia and other comorbidities in all settings and at all stage of the disease? | 4 | 2 | 62.8% | 24 |
| 5. How can distress that is not related to pain be best assessed and managed in palliative patients with dementia that affect communication? What are the best approaches to reducing distress such as life story, pictures, reminiscence? How can sensory and environmental triggers be identified for people with dementia and what education around coping strategies could support and reduce distress? | 4.5 | 1 | 81.4% | 3 |
| 6. What are the best ways to diagnose and treat delirium, agitation, distress, and restlessness in people with dementia at the end of life? Which sedative drugs (such as midazolam, haloperidol and levomepromazine) are most beneficial and best in terms of side-effects? Do these drugs have an effect on other symptoms? | 4 | 2 | 69.8% | 9 |
| 7. What are the best ways to recognise and treat depression, anxiety and low mood in people with  dementia who are approaching end of life, particularly those with learning disabilities and serious  mental ill health? What are the pros and cons of different psychotherapeutic interventions,  including drug therapies, music therapies, animal/pet therapies, and when is the best time to  provide them? | 4 | 2 | 67.5% | 12 |
| 8. What are the best ways to provide emotional and social support for people with dementia and  families? | 3.5 | 2 | 58.1% | 29 |
| 9. What are the best ways to assess faith affiliation and involvement, sources of support, and  spiritual needs and wellbeing (such as from proxies – family members and carers) for people with  dementia in all settings and families? | 2.5 | 1.5 | 44.2% | 44 |
| 10. What are the most effective ways of supporting carers of people with dementia living at home? | 4 | 1.5 | 74.4% | 6 |
| 11. What are the best ways to support children and young people or older people and close friends  when someone close to them is dying or has died from dementia? This includes communicating  with them about the diagnosis and dying process, enabling them to talk about their experience  and providing bereavement support. | 3.5 | 2 | 53.5% | 33 |
| 12. What information and training do carers and families need to provide the best care for their loved one with dementia who is dying, including training for giving medicines at home, carers support and family education? | 4 | 2 | 67.5% | 12 |
| 13. How best to assess and manage family caregiver burden? What are the support needs of family  caregivers of people with dementia, including shared decision making, before and after  bereavement? | 3.5 | 2 | 67.5% | 12 |
| 14. How can it be ensured that staff, including healthcare assistants, are adequately trained to deliver palliative care for people with dementia, no matter where the care is being delivered? Does increasing the number of staff increase the quality of care provided in all settings? To what extent does funding affect these issues? How can cultural competence of palliative care staff be improved? | 4 | 1 | 83.7% | 2 |
| 15. What are the most effective methods to improve the awareness and attitudes of all health and  social care professionals towards people with dementia in all settings and improve their understanding of the challenges faced by carers and families? | 3.5 | 1.5 | 62.8% | 24 |
| 16. How can access to palliative care services be improved for people with dementia regardless of  where they are in the UK (e.g., information and signposting for people to access)? | 2.5 | 2 | 48.8% | 43 |
| 17. How can people with dementia who live alone and do not have any friends or family nearby receive adequate palliative care, particularly if they wish to stay in their homes? | 3.5 | 2 | 72.1% | 8 |
| 18. Are outcomes (e.g., symptom control and incidental prolonging of life) better for terminally ill people with dementia the sooner palliative care is introduced, and services are accessed? | 3.5 | 2 | 51.1% | 41 |
| 19. How could we develop and deliver culturally appropriate palliative care for people with dementia in underrepresented groups such as BAME or minority groups and communities including displaced persons, prisoners, homeless, and LGBTQ+? | 3.5 | 2 | 65.1% | 19 |
| 20. How do we provide culturally competent care to the LGBTQ+ older people with dementia? How  can we assess their needs and provide support for their partners? | 3.5 | 1 | 48.9% | 42 |
| 21. What are the stigma and language barriers regarding dementia and older people in end-of-life  care? How can we tackle stereotypes of dementia? | 3.5 | 2 | 55.8% | 31 |
| 22. What are the best ways to determine a person’s palliative care needs, then initiate and deliver this care for people with dementia to address what matters to each patient and meet individual needs? | 4 | 2 | 53.5% | 33 |
| 23. What are the best ways for healthcare professionals to communicate with people with dementia, carers and families about their diagnosis and explain the dying process compassionately and honestly? Can literature, including leaflets, be helpful? Who is the best person to provide this  information and communication? | 4 | 2 | 65.1% | 19 |
| 24. What are the best models of shared decision-making including people with dementia and family  caregivers as partners? | 3 | 1 | 55.8% | 31 |
| 25. How do we identify preferences with regard to place of care, and which factors should be  considered in decisions on place of care (such as best interest, safety, and family caregiver burden  issues) for people with dementia? | 3 | 1.5 | 51.2% | 39 |
| 26. What are the appropriate end-of-life care tools or standardised measures for use in people with  dementia? What core outcome set should be measured in this area of research? | 2.5 | 1 | 44.2% | 44 |
| 27. What are the needs and priorities of palliative and end of life care for young persons with dementia when diagnosed at working age and their carers? | 3.5 | 1.5 | 74.5% | 5 |
| 28. What are the best ways to facilitate communication across services and between healthcare  professionals for people with dementia, including effective IT systems, shared records,  multidisciplinary team approach with input from other allied health professionals and support  roles, and remote technology (e.g., apps, wearable devices to facilitate care and safety)? | 4 | 1.5 | 60.5% | 27 |
| 29. What are the best ways to make sure there is continuity of care for people with dementia at the  end of life, considering relevant agencies and key clinicians. Does this improve quality of  palliative care and who will coordinate this? Would having a designated case coordinator improve  this process? | 3 | 2 | 62.8% | 24 |
| 30. What are the best ways of providing palliative care outside of working hours for people with  dementia to avoid crises and help patients to stay in their place of choice? (This includes symptom  management, counselling and advice, GP visits and 24-hour support, for patients, carers, and  families.) | 3 | 2 | 65.1% | 19 |
| 31. What interventions are most effective for managing behaviours that others find challenging in people with dementia? What can we do to acknowledge the feelings of those with dementia and  understand how their feelings are affected by reactions of their carers in their own home or  approaches by care home support workers which may cause a reactive behaviour that either the  individual and/ or their support network have difficulty managing? | 3.5 | 2 | 67.4% | 18 |
| 32. What are the benefits of advance care planning and other approaches to listening to and incorporating the preferences of people with dementia (or listening to caregivers, considering capabilities of patients)? Who should implement this and when? | 2.5 | 1.5 | 69.8% | 9 |
| 33. What are the most effective components of care that keep a person with dementia as independent as they can be at all stages of the disease in all care settings? | 2.5 | 2 | 65.1% | 19 |
| 34. What aspects of quality care by carers/care staff improve outcomes for people with dementia?  How can high quality care be implemented for people with dementia in all care settings including  care homes and hospitals? | 2.5 | 2 | 60.5% | 27 |
| 35. What are the most effective design features for producing dementia friendly environments at both the housing and neighbourhood levels? (This question includes the consideration of  design/adaptation of housing e.g., signage, scenery, space & light; for different cultural and ethnic minority groups; design features that best support and enable people with dementia and carers to  maintain quality of life, manage symptoms, minimise behavioural problems, slow progression of  the illness and allow people to stay in their own homes for longer.) | 3 | 1.5 | 53.5% | 33 |
| 36. What are the most effective features of care homes in maintaining safe and secure environments for people with dementia? | 2.5 | 2 | 53.5% | 33 |
| 37. What are the best models and approaches to providing palliative care for people with dementia in an acute setting, such as a hospital? | 3.5 | 2 | 67.5% | 12 |
| 38. What are the best models and approaches to providing palliative care for people with dementia in care homes, including symptom relief, emotional and spiritual support for patients, carers and  families? Is this beneficial to the palliative pathway? How best can we provide strategies in this  setting to meet all individual needs? | 3.5 | 1.5 | 67.5% | 12 |
| 39. What are the best models and approaches to providing palliative care for people with dementia at home and how can home care be maintained as long as possible? Does good coordination of services affect this? | 4 | 2 | 69.8% | 9 |
| 40. What are the best care packages for people with dementia, carers, family, and staff which  combine health care and social care? How do we ensure the best care package for people with  dementia and those supporting them taking individual prognosis into consideration? | 3 | 1.5 | 53.5% | 33 |
| 41. What are the best approaches to care for people with dementia, their carers and families in all  care settings? What challenges are faced and what adaptations are to be made to address cultural  or religious differences? | 3 | 1 | 51.2% | 39 |
| 42. How do all sectors (including community support e.g., police) work together to deliver the  integrated care for people with dementia? | 2.5 | 1.5 | 65.1% | 19 |
| 43. What are the signs that a person with dementia will die in the next few days and how can  detection of these signs be improved? How can families be made aware? When is the optimal time  to initiate palliative care? | 3.5 | 2 | 67.5% | 12 |
| 44. When is the optimal time to move a person with dementia into a care home setting? Questions  include whether it is better to move a person with dementia to a care home at an earlier or later  stage of the disease for outcomes including quality of life, trauma to the person with dementia,  behaviour, physical function, costs; consideration of conditions for staff (salaries, career structure  and training) in improving care; monitoring of standards of care, characteristics of a good care  home. | 2.5 | 1 | 53.5% | 33 |
| 45. Is there an appropriate time to withdraw artificial hydration and nutrition (e.g., a drip) and IV  antibiotics and how can this be done individualised, sensitively and consensually for people with  dementia? What is the best way to communicate with the carers and family about this process? | 3.5 | 2 | 58.1% | 29 |
